# Genetics of autism spectrum disorder: an umbrella review of systematic reviews and meta-analyses

**DOI:** 10.1038/s41398-022-02009-6

**Published:** 2022-06-15

**Authors:** Shuang Qiu, Yingjia Qiu, Yan Li, Xianling Cong

**Affiliations:** 1 Department of Biobank, China-Japan Union Hospital, Jilin University, Changchun, 130033 Jilin, China; 2 China-Japan Union Hospital, Jilin University, Changchun, 130033 Jilin, China; 3 Department of Epidemiology, School of Public Health, Beihua University, Jilin, 132013 Jilin, China

**Keywords:** Autism spectrum disorders, Genetics

## Abstract

Autism spectrum disorder (ASD) is a class of neurodevelopmental conditions with a large epidemiological and societal impact worldwide. To date, numerous studies have investigated the associations between genetic variants and ASD risk. To provide a robust synthesis of published evidence of candidate gene studies for ASD, we performed an umbrella review (UR) of meta-analyses of genetic studies for ASD (PROSPERO registration number: CRD42021221868). We systematically searched eight English and Chinese databases from inception to March 31, 2022. Reviewing of eligibility, data extraction, and quality assessment were performed by two authors. In total, 28 of 5062 retrieved articles were analyzed, which investigated a combined 41 single nucleotide polymorphisms (SNPs) of nine candidate genes. Overall, 12 significant SNPs of *CNTNAP2*, *MTHFR*, *OXTR*, *SLC25A12*, and *VDR* were identified, of which associations with suggestive evidence included the C677T polymorphism of *MTHFR* (under allelic, dominant, and heterozygote models) and the rs731236 polymorphism of *VDR* (under allelic and homozygote models). Associations with weak evidence included the rs2710102 polymorphism of *CNTNAP2* (under allelic, homozygote, and recessive models), the rs7794745 polymorphism of *CNTNAP2* (under dominant and heterozygote models), the C677T polymorphism of *MTHFR* (under homozygote model), and the rs731236 polymorphism of *VDR* (under dominant and recessive models). Our UR summarizes research evidence on the genetics of ASD and provides a broad and detailed overview of risk genes for ASD. The rs2710102 and rs7794745 polymorphisms of *CNTNAP2*, C677T polymorphism of *MTHFR*, and rs731236 polymorphism of *VDR* may confer ASD risks. This study will provide clinicians and healthcare decision-makers with evidence-based information about the most salient candidate genes relevant to ASD and recommendations for future treatment, prevention, and research.

## Introduction

Autism spectrum disorder (ASD) is a group of neurodevelopmental conditions characterized by early-onset dysfunctions in communication, impairments in social interaction, and repetitive and stereotyped behaviors and interests [1]. Patients develop ASD-related symptoms when they are 12−18 months of age, and diagnosis is generally made at the age of 2 years [2]. In 2010, 52 million people had been diagnosed with ASD worldwide, which was equivalent to a population prevalence of 7.6 per 1000 or 1 in 132 persons [3]. ASD is the leading cause of disability in children under 5 years, and people with ASD may require high levels of support, which is costly and thus leads to substantial economic, emotional, and physical burdens on affected families [3].

Due to the lack of clinical and epidemiological evidence for an ASD cure, researchers have focused on better understanding ASD and advancing risk prediction and prevention [3]. The causes of ASD are complex and multifactorial, with several associated genes and environmental risk factors [4]. A previous umbrella review (UR) of environmental risk factors for ASD showed that several maternal factors, including advanced age (≥35 years), chronic hypertension, preeclampsia, gestational hypertension, and being overweight before or during pregnancy, were significantly associated with ASD risk, without any signs of bias [5, 6]. Accumulating twin- and family based studies further indicate that genetic factors play critical roles in ASD, such that the concordance rate among monozygotic twins is higher (60–90%) than that among dizygotic twins (0–30%) [7, 8]. The heritability of ASD has been estimated to be 50%, indicating that genetic factors are the main contributors to the etiology of ASD [8].

To date, numerous studies investigating the association between genetic variants and ASD risk have been published [9–11]. Most of these studies focused on identifying single nucleotide polymorphisms (SNPs) of candidate genes associated with ASD risk. However, these SNP studies had small sample sizes and, therefore, low statistical power to demonstrate statistically significant effects of low-risk susceptibility genes, leading to inconsistent conclusions. Although meta-analyses have been conducted to resolve this problem, single SNPs or genes have usually been investigated.

An UR collects and evaluates multiple systematic reviews and meta-analyses conducted on a specific research topic, provides a robust synthesis of published evidence, and considers the importance of effects found over time [12]. In addition, the results of UR studies may increase the predictive power with more precise estimates [13]. Thus, we aimed to perform an UR study of all the systematic reviews and meta-analyses that have been published, assessing candidate genes associated with ASD risk. This study will provide clinicians and healthcare decision-makers with evidence-based information about candidate genes of ASD and recommendations for future prevention and research in less time than would otherwise be required to locate and examine all relevant research individually.

## Methods

### Literature search strategy and eligibility criteria

We systematically searched the PubMed, EMBASE, PsycINFO, Web of Science, Cochrane Library, China National Knowledge Infrastructure, Sinomed, and Wanfang databases from inception to March 31, 2022. The databases were searched using the following strategy: (autis* [All Fields] OR autism* [All Fields] OR autistic* [All Fields] OR ASD [All Fields] OR autism spectrum disorder* [All Fields] OR PDD-NOS [All Fields] OR PDDNOS [All Fields] OR unspecified PDD [All Fields] OR PDD [All Fields] OR pervasive developmental disorder* [All Fields] OR pervasive developmental disorder not otherwise specified [All Fields] OR Asperger* [All Fields] OR Asperger* syndrome [All Fields]) AND (gene* [All Fields] OR genom* [All Fields]) AND (systematic review [All Fields] OR meta-analysis [All Fields]). Authors S. Qiu and Y. Qiu independently conducted literature searches for potential articles included in this review. The references of the relevant articles were manually searched to identify and incorporate eligible studies.

We included meta-analyses of family based and case-control studies that examined associations between ASD and potential risk genes. We only included meta-analyses that reported either effect estimates of individual study or the data necessary to calculate these estimates. We excluded meta-analyses if (1) risk genes were used for screening, diagnostic, or prognostic purposes; (2) a study examined ASD as a risk factor for other medical conditions; (3) a study included fewer than three original studies investigating the association between risk genes and ASD; and (4) a study with missing information after the corresponding author, whom we contacted through email, failed to provide the required information. All articles retrieved were first organized in the reference manager software (Endnote 9, Clarivate Analytics, New York, NY, USA), and duplicates were deleted. S. Qiu and Y. Qiu chose eligible articles by screening the titles, abstracts, and full article texts independently. Disagreements were resolved through a discussion with a third investigator (Y. Li) until a consensus was reached.

### Data extraction and quality assessment

From each eligible meta-analysis, we extracted the first author, publication year, genetic risk factors examined, number of studies, number of ASD cases and participants, study-specific relative risk estimates (odds ratio [*OR*]) with the corresponding 95% confidence interval (*CI*), sample size of cases and controls, genotype and allele counts, and individual study designs (case-control, family based or mixed [case-control and family based]). We used the ‘assessment of multiple systematic reviews’ tool, consisting of 11 items, to assess the methodological quality of the meta-analyses [14]. Data extraction and quality assessment were independently conducted by S. Qiu and Y. Qiu. Disagreements were resolved via a discussion with a third investigator (Y. Li) until a consensus was reached.

### Data analysis

In agreement with previous URs, we performed a statistical analysis using a series of tests that were previously developed and reproduced [13, 15, 16]. If more than one meta-analysis on the same research question was eligible, the most recent meta-analysis was retained for the main analysis. For each eligible meta-analysis, we calculated the summary-effect size with 95% *CI* [17]. We also calculated the 95% prediction interval (*PI*) to explain the between-study heterogeneity and to assess the uncertainty of a new study [18, 19]. Heterogeneity between studies was assessed using the Chi-squared test based Q-statistic and quantified using the *I*^*2*^-statistic [20, 21]. If there was no substantial statistical heterogeneity (*P* > 0.10, *I*^*2*^ ≤ 50%), data were pooled using a fixed-effect model; otherwise, heterogeneity was evaluated using a random-effect model [22]. The Hardy–Weinberg equilibrium (HWE) of meta-analyses in the control group was analyzed using Chi-squared tests. Additionally, small-study effects were evaluated using Egger’s regression asymmetry test. *P*-values < 0.10 were considered to indicate the presence of small-study effects [23, 24]. The Chi-squared test was used to assess the presence of excess significance, which evaluated whether the observed number of studies with significant results (*P* < 0.05) was greater than the expected number [22, 25]. All statistical analyses were performed using RStudio 3.6.2. Statistical significance was set at *P* < 0.05, except where otherwise specified.

### Determining the credibility of evidence

In line with previous URs, we categorized the strength of the evidence of risk genes for ASD into five levels: convincing (class I), highly suggestive (class II), suggestive (class III), weak (class IV), and not significant [5, 26–28]. Criteria for the level of evidence included the number of ASD cases, *P*-values by random effects model, small-study effects, excess significance bias, heterogeneity (*I²*), and 95% *CI*.

This review was prospectively registered with PROSPERO (registration number: CRD42021221868).

## Results

### Description of eligible meta-analyses

**Fig. 1:**
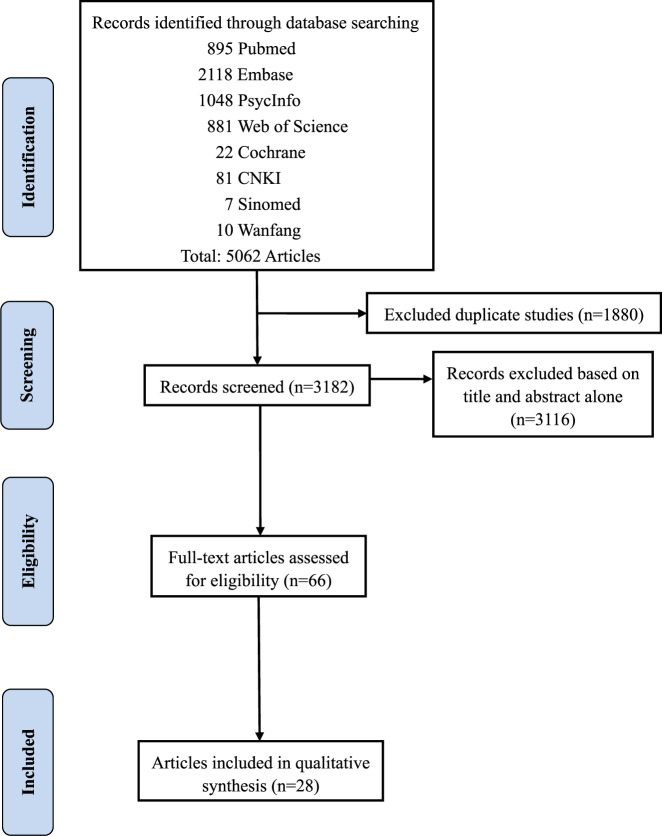
Flow chart of literature identification and selection.

A total of 5062 articles were identified through an initial search. After removing duplicates, the titles and abstracts of 3182 articles were screened for eligibility. Of the remaining 66 articles that were reviewed in full, 28 eligible articles were selected for data extraction (Fig. 1).

The characteristics of the selected studies are presented in Table 1. Of the 28 included reviews, eight were on methylenetetrahydrofolate reductase (*MTHFR*) [29–36]; four each on solute carrier family 6 member 4 (*SLC6A4*) [37–40] and contactin associated protein 2 (*CNTNAP2*) [41–44]; three each on oxytocin receptor (*OXTR*) [45–47] and reelin (*RELN*) [48–50]; two each on gamma-aminobutyric acid type A receptor subunit beta3 (*GABRB3*) [51, 52], solute carrier family 25 member 12 (*SLC25A12*) [53, 54], and vitamin D receptor (*VDR*) [55, 56]; and one on catechol-o-methyltransferase (*COMT*) [39] (one meta-analysis was on both *COMT* and *SLC6A4*). These studies were published from 2008 to 2021 and considered the associations between 41 SNPs in nine candidate genes and ASD risk. For quality assessment, 22 articles that scored 5−8 were rated as ‘moderate quality’, and six that scored < 5 were rated as ‘low quality’. Seventeen studies (60.7%) performed the HWE check (Table 1). With respect to the study design, 14 (64.3%) studies synthesized case-control studies, two (7.1%) included family based studies, and eight (28.6%) used both case-control and family based studies (Table 1).

**Table 1:** Information on meta-analyses included in the umbrella review.

Genes	Studies	Study design	HWE check	AMSTAR
*CNTNAP2*	Uddin et al. [44]	case control	Yes	5
*CNTNAP2*	Wang et al. [43]	case control	–	5
*CNTNAP2*	Werling et al. [41]	case control&family based	–	3
*CNTNAP2*	Zhang et al. [42]	case control&family based	–	5
*COMT*	Yang et al. [39]	case control	Yes	6
*GABRB3*	Mahdavi et al. [51]	case control	Yes	5
*GABRB3*	Noroozi et al. [52]	case control	Yes	5
*MTHFR*	Li et al. [33]	case control	Yes	5
*MTHFR*	Li et al. [34]	case control	Yes	5
*MTHFR*	Pu et al. [29]	case control	Yes	5
*MTHFR*	Rai [30]	case control	Yes	3
*MTHFR*	Razi et al. [32]	case control	Yes	6
*MTHFR*	Sadeghiyeh et al. [31]	case control	Yes	5
*MTHFR*	Wang and Wu [35]	case control	Yes	6
*MTHFR*	Zhanget al. [36]	case control	Yes	6
*OXTR*	Kranz et al. [46]	family based	–	2
*OXTR*	LoParo and Waldman [45]	case control&family based	–	5
*OXTR*	Zhou [47]	case control	–	6
*RELN*	Chen [49]	case control	–	4
*RELN*	Hernández-García (2020) [50]	case control	–	3
*RELN*	Wang [48]	case control&family based	Yes	5
*SLC25A12*	Aoki and Cortese [53]	case control&family based	–	4
*SLC25A12*	Liu et al. [54]	case control&family based	Yes	7
*SLC6A4*	Huang and Santangelo [37]	family based	Yes	5
*SLC6A4*	Mo et al. [38]	case control&family based	–	5
*SLC6A4*	Wang et al. [40]	case control	–	6
*SLC6A4*	Yang et al. [39]	case control&family based	Yes	6
*VDR*	Sun [55]	case control	Yes	6
*VDR*	Yang and Wu [56]	case control	Yes	5

*HWE* Hardy–Weinberg equilibrium, – no data/data not complete.

### Summary-effect sizes and significant findings

The results of the associations between the 41 SNPs and ASD risks reported in the meta-analyses are presented in Table 2 under five different genetic models: allelic model (mutant allele vs. wild-type allele), dominant model (mutant homozygote + heterozygote vs. wild-type homozygote), heterozygote model (heterozygote vs. wild-type homozygote), homozygote model (mutant homozygote vs. wild-type homozygote), and recessive model (mutant homozygote vs. wild-type homozygote + heterozygote).

**Table 2:** Results of meta-analyses included in the umbrella review.

Studies	Genes	SNPs	Number of studies	Allelic model	Dominant model	Heterozygote model	Homozygote model	Recessive model
				*OR* (95%*CI*)	*OR* (95%*CI*)	*OR* (95%*CI*)	*OR* (95%*CI*)	*OR* (95%*CI*)
Uddin et al. [44]	*CNTNAP2*	rs2710102	5	**0.85 (0.73–0.98)**	0.88 (0.68–1.14)	0.96 (0.74–1.26)	**0.67 (0.47–0.95)**	**0.72 (0.56–0.91)**
Wang et al. [43]	*CNTNAP2*	rs2710102	7	1.00 (0.84–1.18)	–	–	–	0.98 (0.77–1.07)
Werling et al. [41]	*CNTNAP2*	rs2710102	5	1.03 (0.98–1.08)	–	–	–	–
Zhang et al. [42]	*CNTNAP2*	rs2710102	7	0.99 (0.94–1.03)	–	–	–	–
Uddin et al. [44]	*CNTNAP2*	rs7794745	8	1.21 (0.97–1.51)	**1.30 (1.11–1.52)**	**1.28 (1.08–1.50)**	1.49 (0.78–2.86)	1.30 (0.69–2.44)
Werling et al. [41]	*CNTNAP2*	rs7794745	6	1.02 (0.99–1.05)	–	–	–	–
Zhanget al. [42]	*CNTNAP2*	rs7794745	8	1.00 (0.90–1.12)	–	–	–	–
Yang et al. [39]	*COMT*	rs4680	4	0.97 (0.84–1.13)	–	–	–	–
Mahdavi et al. [51]	*GABRB3*	rs1426217	3	1.13 (0.64–2.00)	–	–	–	–
Noroozi et al. [52]	*GABRB3*	rs20317	3	0.92 (0.78–1.08)	0.97 (0.65–1.44)	0.86 (0.50–1.47)	1.07 (0.74–1.55)	1.09 (0.82–1.46)
Mahdavi et al. [51]	*GABRB3*	rs2081648	4	0.84 (0.41–1.72)	–	–	–	–
Noroozi et al. [52]	*GABRB3*	rs4906902	5	1.04 (0.92–1.17)	0.98 (0.83–1.16)	0.96 (0.82–1.13)	0.94 (0.71–1.24)	0.94 (0.72–1.23)
Li et al. [33]	*MTHFR*	A1298C	9	1.17 (0.91–1.50)	1.19 (0.87–1.64)	1.11 (0.82–1.50)	1.31 (0.82–2.09)	1.17 (0.76–1.78)
Pu et al. [29]	*MTHFR*	A1298C	5	0.86 (0.68–1.08)	0.93 (0.70–1.23)	0.98 (0.68–1.43)	0.79 (0.59–1.07)	0.73 (0.56–0.97)
Razi et al. [32]	*MTHFR*	A1298C	8	1.18 (0.86–1.63)	1.17 (0.78–1.75)	1.19 (0.80–1.76)	1.00 (0.61–1.64)	0.77 (0.40–1.49)
Sadeghiyeh et al. [31]	*MTHFR*	A1298C	7	0.94 (0.77–1.16)	0.98 (0.74–1.30)	1.04 (0.75–1.44)	0.92 (0.69–1.21)	0.83 (0.64–1.08)
Li et al. [33]	*MTHFR*	C677T	15	**1.63 (1.30–2.05)**	**1.82 (1.39–2.37)**	**1.66 (1.31–2.11)**	**2.03 (1.33–3.09)**	**1.59 (1.14–2.22)**
Li et al. [34]	*MTHFR*	C677T	6	**1.88 (1.15–3.08)**	**1.96 (1.18–3.25)**	**1.68 (1.11–2.55)**	**2.31 (1.23–4.34)**	**1.93 (1.09–3.40)**
Pu et al. [29]	*MTHFR*	C677T	8	**1.42 (1.09–1.85)**	**1.56 (1.12–2.18)**	**1.48 (1.09–2.00)**	**1.86 (1.08–3.20)**	**1.56 (1.12–2.18)**
Rai [30]	*MTHFR*	C677T	13	**1.48 (1.18–1.86)**	1.70 (0.96–2.90)	**1.60 (1.20–2.10)**	**1.84 (1.12–3.02)**	**1.50 (1.00–2.20)**
Razi [32]	*MTHFR*	C677T	17	**1.37 (1.08–1.74)**	**1.47 (1.13–1.93)**	**1.45 (1.13–1.85)**	1.40 (0.87–2.27)	1.14 (0.79–1.64)
Sadeghiyeh et al. [31]	*MTHFR*	C677T	18	**1.64 (1.30–2.08)**	**1.60 (1.12–2.30)**	**1.51 (1.09–2.10)**	**1.99 (1.29–3.06)**	**1.48 (1.06–2.08)**
Wang and Wu [35]	*MTHFR*	C677T	14	**1.63 (1.20–2.22)**	**1.75 (1.28–2.38)**	**1.56 (1.24–1.98)**	**1.60 (1.06–2.41)**	1.33 (0.93–1.92)
Zhang [36]	*MTHFR*	C677T	16	**1.80 (1.30–2.48)**	**1.96 (1.40–2.74)**	**1.77 (1.34–2.33)**	**1.80 (1.16–2.78)**	1.42 (0.98–2.07)
LoParo and Waldman [45]	*OXTR*	rs1042778	6	0.97 (0.87–1.09)	–	–	–	–
LoParo and Waldman [45]	*OXTR*	rs11706648	4	1.02 (0.89–1.18)	–	–	–	–
LoParo and Waldman [45]	*OXTR*	rs2254298	6	1.15 (0.93–1.43)	–	–	–	–
Zhou [47]	*OXTR*	rs2254298	5	1.06 (0.81–1.38)	1.06 (0.85–1.31)	1.03 (0.82–1.29)	1.26 (0.79–2.02)	1.25 (0.79–1.97)
LoParo and Waldman [45]	*OXTR*	rs2268490	4	1.13 (0.93–1.34)	–	–	–	–
LoParo and Waldman [45]	*OXTR*	rs2268491	6	**1.19 (1.05–1.36)**	–	–	–	–
LoParo and Waldman [45]	*OXTR*	rs2268493	4	0.98 (0.71–1.33)	–	–	–	–
LoParo and Waldman [45]	*OXTR*	rs2268495	6	0.97 (0.78–1.21)	–	–	–	–
Zhou [47]	*OXTR*	rs2301261	3	1.00 (0.62–1.63)	–	–	–	–
LoParo and Waldman [45]	*OXTR*	rs237885	8	0.96 (0.85–1.08)	–	–	–	–
LoParo and Waldman [45]	*OXTR*	rs237887	6	**0.88 (0.79–0.98)**	–	–	–	–
LoParo and Waldman [45]	*OXTR*	rs237888	4	1.17 (0.92–1.50)	–	–	–	–
Kranz [46]	*OXTR*	rs237889	4	**1.12 (1.01–1.24)**	–	–	–	–
LoParo and Waldman [45]	*OXTR*	rs237894	4	1.03 (0.84–1.27)	–	–	–	–
LoParo and Waldman [45]	*OXTR*	rs237895	4	1.21 (0.98–1.48)	–	–	–	–
Kranz et al. [46]	*OXTR*	rs237897	4	1.05 (0.88–1.25)	–	–	–	–
LoParo and Waldman [45]	*OXTR*	rs4684302	4	0.87 (0.64–1.23)	–	–	–	–
LoParo and Waldman [45]	*OXTR*	rs4686301	4	1.15 (0.92–1.43)	–	–	–	–
LoParo and Waldman [45]	*OXTR*	rs53576	5	0.91 (0.76–1.09)	–	–	–	–
Zhou [47]	*OXTR*	rs53576	4	0.91 (0.80–1.02)	0.84 (0.59–1.19)	0.79 (0.55–1.13)	0.91 (0.64–1.29)	0.93 (0.54–1.60)
LoParo and Waldman [45]	*OXTR*	rs7632287	4	**1.44 (1.23–1.68)**	–	–	–	–
Chen et al. [49]	*RELN*	rs2229864	4	1.01 (0.83–1.24)	1.08 (0.84–1.38)	–	–	–
Hernández-García et al. [50]	*RELN*	rs2229864	4	–	–	–	–	0.75 (0.48–1.16)
Chen et al. [49]	*RELN*	rs362691	5	0.88 (0.70–1.10)	0.87 (0.68–1.11)	–	–	–
Hernández-García et al. [50]	*RELN*	rs362691	6	–	–	–	–	1.03 (0.77–1.38)
Wang et al. [48]	*RELN*	rs362691	3	0.80 (0.44–1.46)	–	–	–	–
Wang et al. [48]	*RELN*	rs362691	5	0.82 (0.61–1.10)	–	–	–	–
Chen et al. [49]	*RELN*	rs607755	3	0.73 (0.53–1.02)	0.76 (0.48–1.20)	–	–	–
Chen et al. [49]	*RELN*	rs736707	5	0.90 (0.67–1.20)	0.87 (0.57–1.33)	–	–	–
Hernández-García et al. [50]	*RELN*	rs736707	6	–	–	–	–	1.02 (0.76–1.37)
Wang et al. [48]	*RELN*	rs736707	6	1.11 (0.80–1.54)	–	–	–	–
Aoki and Cortese [53]	*SLC25A12*	rs2056202	11	**1.21 (1.04–1.41)**	–	–	–	–
Aoki and Cortese [53]	*SLC25A12*	rs2056202	5	1.07 (0.85–1.34)	–	–	–	–
Aoki and Cortese [53]	*SLC25A12*	rs2056202	6	**1.27 (1.04–1.54)**	–	–	–	–
Liu et al. [54]	*SLC25A12*	rs2056202	8	**0.81 (0.71–0.92)**	–	–	–	–
Liu et al. [54]	*SLC25A12*	rs2056202	5	**0.78 (0.67–0.90)**	–	–	–	–
Liu et al. [54]	*SLC25A12*	rs2056202	4	0.99 (0.80–1.22)	–	–	–	–
Aoki and Cortese [53]	*SLC25A12*	rs2292813	10	**1.19 (1.05–1.35)**	–	–	–	–
Aoki and Cortese [53]	*SLC25A12*	rs2292813	3	0.90 (0.59–1.36)	–	–	–	–
Aoki and Cortese [53]	*SLC25A12*	rs2292813	7	**1.22 (1.08–1.38)**	–	–	–	–
Liu et al. [54]	*SLC25A12*	rs2292813	7	**0.75 (0.65–0.87)**	–	–	–	–
Liu et al. [54]	*SLC25A12*	rs2292813	6	**0.75 (0.63–0.88)**	–	–	–	–
Huang et al. [37]	*SLC6A4*	5-HTTLPR	13	1.03 (0.84–1.27)	–	–	–	–
Huang et al. [37]	*SLC6A4*	5-HTTLPR	14	1.05 (0.88–1.25)	–	–	–	–
Mo et al. [38]	*SLC6A4*	5-HTTLPR	6	1.19 (0.83–1.72)	–	–	–	–
Mo et al. [38]	*SLC6A4*	5-HTTLPR	19	1.07 (0.92–1.25)	–	–	–	–
Mo et al. [38]	*SLC6A4*	5-HTTLPR	25	1.10 (0.95–1.26)	–	–	–	–
Wang et al. [40]	*SLC6A4*	5-HTTLPR	11	1.13 (0.95–1.34)	1.11 (0.91–1.35)	–	1.20 (0.82–1.78)	1.08 (0.73–1.58)
Yang et al. [39]	*SLC6A4*	5-HTTLPR	18	1.04 (0.89–1.21)	–	–	–	–
Yang et al. [39]	*SLC6A4*	5-HTTLPR	6	1.19 (0.86–1.65)	–	–	–	–
Huang et al. [37]	*SLC6A4*	STin2 VNTR	8	1.13 (0.82–1.56)	–	–	–	–
Sun [55]	*VDR*	rs11568820	4	1.05 (0.89–1.23)	1.03 (0.83–1.27)	0.99 (0.79–1.24)	1.12 (0.78–1.60)	1.15 (0.82–1.62)
Yang and Wu [56]	*VDR*	rs11568820	3	1.12 (0.92–1.37)	1.06 (0.67–1.66)	0.99 (0.60–1.64)	1.19 (0.78–1.81)	1.21 (0.82–1.80)
Sun and Wu [55]	*VDR*	rs1544410	5	1.07 (0.92–1.24)	1.04 (0.84–1.30)	1.00 (0.79–1.25)	1.16 (0.84–1.61)	1.17 (0.89–1.52)
Yang and Wu [56]	*VDR*	rs1544410	5	1.07 (0.92–1.24)	1.02 (0.82–1.28)	0.96 (0.76–1.21)	1.20 (0.86–1.67)	1.20 (0.92–1.58)
Sun [55]	*VDR*	rs2228570	7	1.09 (0.96–1.24)	1.01 (0.84–1.21)	0.93 (0.77–1.13)	**1.39 (1.04–1.87)**	**1.36 (1.05–1.75)**
Yang and Wu [56]	*VDR*	rs2228570	4	0.95 (0.80–1.12)	0.86 (0.67–1.10)	0.81 (0.63–1.05)	0.99 (0.69–1.44)	1.06 (0.79–1.43)
Sun [55]	*VDR*	rs731236	6	**1.30 (1.12–1.49)**	**1.30 (1.08–1.57)**	1.20 (0.86–1.67)	**1.74 (1.26–2.41)**	**1.61 (1.19–2.19)**
Yang and Wu [56]	*VDR*	rs731236	3	**1.33 (1.09–1.61)**	1.26 (0.79–2.01)	1.10 (0.60–2.01)	**2.09 (1.34–3.25)**	**1.96 (1.30–2.96)**
Sun [55]	*VDR*	rs7975232	3	**0.82 (0.68–0.99)**	0.74 (0.54–1.02)	0.76 (0.54–1.07)	0.53 (0.22–1.28)	0.74 (0.40–1.34)
Yang and Wu [56]	*VDR*	rs7975232	3	**0.82 (0.68–0.99)**	0.74 (0.54–1.02)	0.76 (0.54–1.07)	0.53 (0.22–1.28)	0.74 (0.40–1.34)

– no data/data not complete.

Only one meta-analysis on the rs2710102 polymorphism of *CNTNAP2* showed that the polymorphism was associated with ASD susceptibility in allelic, homozygote, and recessive models [44]. This meta-analysis also found that the rs7794745 polymorphism of *CNTNAP2* was associated with an increased risk of ASD in dominant and heterozygote models [44].

All four meta-analyses reported no significant association between the A1298C polymorphism of *MTHFR* and ASD risk. All eight meta-analyses on the C677T polymorphism of *MTHFR* showed that the polymorphism was associated with ASD susceptibility in allelic and heterozygote models [29–36]. Seven meta-analyses found that the C677T polymorphism was associated with an increased risk of ASD in dominant [29, 31–36] and homozygote [29–31, 33–36] models. Five meta-analyses found that the C677T polymorphism was associated with an increased risk of ASD in the recessive model [29–31, 33, 34].

For *OXTR*, 19 SNPs were summarized. LoParo et al. [45] found that the mutant allele of rs2268491, wild-type allele of rs237887, and mutant allele of rs7632287 were risk-inducing SNPs of ASD. In addition, Kranz et al. [46] found that the mutant allele of rs237889 was associated with ASD risk.

Regarding *SLC25A12*, both Aoki et al. [53] and Liu et al. [54] found that the mutant alleles of rs2056202 and rs2292813 significantly increased ASD risk in family-based and mixed studies. We excluded the results of the associations between rs2292813 and ASD risk based on the case-control design reported by Liu et al. [54], as the authors included only two case–control studies.

Sun et al. [55] found that the rs2228570 polymorphism of *VDR* was associated with an increased ASD risk in homozygote and recessive models, while Yang et al. [56] did not find significant associations in any genetic model. Both authors [55, 56] found that the rs731236 polymorphism of *VDR* was significantly associated with ASD risk in allelic, homozygote, and recessive models. Sun et al. [55] found that the rs731236 polymorphism was significantly associated with ASD risk in the dominant model. Both Sun et al. [55] and Yang et al. [56] found that the mutant allele of rs7975232 of *VDR* was significantly associated with a decreased ASD risk (Table 2). There were no significant SNPs in *COMT*, *GABRB3*, *RELN*, and *SLC6A4*.

### Determining the credibility of evidence

When more than one meta-analysis on the same research question was eligible, the most recent one was retained for the main analysis. After comparing the publication year and sample size of each meta-analysis, 11 meta-analyses were retained for further analysis, of which two each study were on *RELN* and *MTHFR*, and one each was on *CNTNAP2*, *COMT*, *GABRB3*, *OXTR*, *SLC25A12*, *SLC6A4*, and *VDR*. We extracted the allele and genotype frequencies of each SNP in case and control groups from the original research for further analysis. However, the allele and genotype frequencies of some SNPs in the compared groups could not be extracted from the original research that did not contain the information, and we could not obtain this information from the corresponding authors of the studies. Finally, we analyzed the data of 20 SNPs with allele frequencies in 10 meta-analyses from 117 original studies and 16 SNPs with genotype frequencies in eight meta-analyses from 101 original studies. Associations were measured using five different genetic models (Tables 3, 4).

**Table 3:** Information on meta-analyses included for further analysis.

Studies	Genes	SNPs	Number of studies	Cases			Controls			
				n	A/B	AA/AB/BB	n	A/B	AA/AB/BB	*P*_value of HWE
Uddin et al. [44]	*CNTNAP2*	rs2710102	5	684	751/617	189/373/122	12563	12204/12922	2964/6276/3323	0.995
		rs7794745	8	1206	936/1476	158/620/428	13191	9404/16978	1682/6040/5469	0.821
Yang et al. [39]	*COMT*	rs4680	4	814	779/849	–	741	690/778	–	…
Noroozi et al. [52]	*GABRB3*	rs20317	3	636	493/779	113/267/256	787	692/882	185/322/280	**<0.001**
		rs4906902	5	1297	729/1865	118/493/686	1423	794/2052	125/544/754	0.061
Li et al. [33]	*MTHFR*	A1298C	9	1961	1182/2740	225/732/1004	2034	1186/2882	209/768/1057	**<0.001**
Zhang et al. [36]	*MTHFR*	C677T	16	2147	1559/2735	290/979/878	2253	1387/3119	259/869/1125	**<0.001**
Zhou [47]	*OXTR*	rs2254298	5	1181	475/1863	–	1790	672/2884	–	…
		rs2301261	3	474	93/855	–	951	179/1723	–	…
		rs53576	4	1081	871/1263	–	1558	1220/1864	–	…
Chen et al. [49]	*RELN*	rs607755	3	298	252/344	52/148/98	270	209/331	44/121/105	0.362
Hernández-García et al. [50]	*RELN*	rs2229864	4	646	969/323	363/243/40	774	1219/329	486/247/41	0.195
		rs362691	6	780	941/619	398/145/237	882	1014/750	419/176/287	**<0.001**
		rs736707	6	868	814/922	201/412/255	1093	995/1191	237/521/335	0.198
Wang et al. [40]	*SLC6A4*	5-HTTLPR	11	930	884/922	243/398/262	1234	1045/1373	282/481/446	**<0.001**
Sun [55]	*VDR*	rs11568820	4	844	478/1210	88/302/454	689	385/993	68/249/372	**0.007**
		rs1544410	5	993	702/1284	161/380/452	904	645/1163	138/369/397	**<0.001**
		rs2228570	7	1107	858/1356	195/468/444	1110	826/1394	163/500/447	0.230
		rs731236	6	1088	664/1512	127/410/551	1020	519/1521	76/367/577	0.099
		rs7975232	3	430	409/451	87/235/108	491	506/476	116/274/101	**0.009**

*A* Mutant allele, *B* Wild-type allele, *HWE* Hardy–Weinberg equilibrium, – no data/data not complete, … cannot calculated.

**Table 4:** Results and assessment of cumulative evidence associations (on random effects model) of genetic variants with risk of ASD.

Studies	Genes	SNPs	Genetic model	Summary model	Summary estimate (95%CI)	*P*_value	Random effects *P*_value	*I*^*2*^ (%)	*P*__heterogeneity_	Egger *P*_value	95%*PI*	Excess Significance (*P*_value)	Credibility of evidence
Uddin et al. [44]	*CNTNAP2*	rs2710102	Allelic	Fixed	**0.849 (0.734–0.981)**	**0.0263**	**0.0263**	0.0	0.711	0.511	**0.734–0.981**	0.843	**Weak**
			Dominant	Fixed	0.883 (0.681–1.144)	0.3455	0.3494	0.0	0.851	0.848	0.681–1.146	0.731	Non-significant
			Heterozygote	Fixed	0.964 (0.736–1.262)	0.7891	0.7896	0.0	0.940	0.946	0.736–1.263	0.700	Non-significant
			Homozygote	Fixed	**0.668 (0.470–0.950)**	**0.0248**	**0.0231**	0.0	0.743	0.403	**0.467–0.946**	0.848	**Weak**
			Recessive	Fixed	**0.715 (0.563–0.909)**	**0.0062**	**0.0061**	0.0	0.632	0.696	**0.562–0.909**	0.890	**Weak**
		rs7794745	Allelic	Random	1.214 (0.974–1.513)	0.0849	0.0849	72.2	**<0.001**	0.487	0.689–2.137	**0.009**	Non-significant
			Dominant	Fixed	**1.300 (1.109–1.523)**	**0.0012**	**0.0081**	32.2	0.171	0.442	0.895–1.914	0.718	**Weak**
			Heterozygote	Fixed	**1.275 (1.081–1.504)**	**0.0039**	**0.0066**	8.8	0.362	0.637	**1.010–1.612**	0.288	**Weak**
			Homozygote	Random	1.490 (0.776–2.859)	0.2309	0.2309	72.5	**<0.001**	0.185	0.277–7.999	**<0.001**	Non-significant
			Recessive	Random	1.301 (0.692–2.444)	0.4140	0.4140	73.4	**<0.001**	0.150	0.253–6.684	**0.001**	Non-significant
Yang et al. [39]	*COMT*	rs4680	Allelic	Random	0.993 (0.779–1.265)	0.9534	0.9534	61.1	**0.053**	0.570	0.642–1.534	**0.041**	Non-significant
Noroozi et al. [52]	*GABRB3*	rs20317	Allelic	Fixed	0.917 (0.781–1.076)	0.2875	0.2878	0.0	0.968	0.605	0.781–1.076	0.712	Non-significant
			Dominant	Random	1.037 (0.699–1.538)	0.8574	0.8574	51.6	0.127	**0.064**	0.557–1.924	0.670	Non-significant
			Heterozygote	Random	1.173 (0.682–2.015)	0.5647	0.5647	70.8	**0.033**	**0.010**	0.436–3.194	**0.014**	Non-significant
			Homozygote	Fixed	0.939 (0.654–1.347)	0.7304	0.7905	31.6	0.232	0.869	0.650–1.357	0.661	Non-significant
			Recessive	Fixed	0.827 (0.624–1.098)	0.1887	0.2492	22.8	0.274	0.467	0.568–1.197	0.733	Non-significant
		rs4906902	Allelic	Fixed	1.042 (0.924–1.175)	0.5010	0.5113	5.1	0.378	0.834	0.844–1.293	0.715	Non-significant
			Dominant	Fixed	1.046 (0.897–1.219)	0.5671	0.6350	25.3	0.253	0.930	0.762–1.436	0.710	Non-significant
			Heterozygote	Fixed	1.034 (0.879–1.215)	0.6873	0.7338	23.2	0.267	0.931	0.750–1.425	0.703	Non-significant
			Homozygote	Fixed	1.066 (0.807–1.408)	0.6547	0.6584	0.0	0.766	0.502	0.806–1.408	0.704	Non-significant
			Recessive	Fixed	1.071 (0.819–1.399)	0.6171	0.6205	0.0	0.930	0.624	0.818–1.399	0.706	Non-significant
Li et al. [33]	*MTHFR*	A1298C	Allelic	Random	1.260 (0.949–1.674)	0.1101	0.1101	85.0	**<0.001**	**0.045**	0.341–5.411	**<0.001**	Non-significant
			Dominant	Random	1.255 (0.895–1.759)	0.1887	0.1887	80.5	**<0.001**	**0.016**	0.298–6.898	**<0.001**	Non-significant
			Heterozygote	Random	1.163 (0.838–1.615)	0.3674	0.3674	75.3	**<0.001**	**0.017**	0.375–4.221	**<0.001**	Non-significant
			Homozygote	Random	1.377 (0.847–2.237)	0.1969	0.1969	72.1	**<0.001**	0.129	0.340–5.947	0.052	Non-significant
			Recessive	Random	1.198 (0.769–1.867)	0.4241	0.4241	70.3	**<0.001**	0.379	0.365–3.993	**0.016**	Non-significant
Zhang et al. [36]	*MTHFR*	C677T	Allelic	Random	**1.799 (1.303–2.483)**	**0.0004**	**0.0004**	83.6	**<0.001**	**0.003**	0.545–5.942	0.072	**Suggestive**
			Dominant	Random	**1.959 (1.402–2.738)**	**<0.0001**	**8.17E-05**	76.2	**<0.001**	**0.004**	0.596–6.435	0.190	**Suggestive**
			Heterozygote	Random	**1.767 (1.343–2.330)**	**<0.0001**	**5.01E-05**	64.2	**<0.001**	**0.004**	0.717–4.365	0.222	**Suggestive**
			Homozygote	Random	**1.795 (1.158–2.782)**	**0.0089**	**0.0089**	64.2	**<0.001**	**0.008**	0.489–6.584	**0.005**	**Weak**
			Recessive	Random	1.424 (0.980–2.069)	0.0634	0.0634	60.0	**0.001**	**0.012**	0.497–4.085	**0.002**	Non-significant
Zhou [47]	*OXTR*	rs2254298	Allelic	Random	1.056 (0.810–1.379)	0.6863	0.6863	65.8	**0.020**	0.158	0.585–1.874	0.381	Non-significant
		rs2301261	Allelic	Random	1.002 (0.617–1.627)	0.9943	0.9943	59.4	**0.085**	0.555	0.459–2.195	0.677	Non-significant
		rs53576	Allelic	Fixed	1.103 (0.978–1.244)	0.1109	0.1341	36.1	0.195	0.273	0.862–1.498	0.776	Non-significant
Chen et al. [49]	*RELN*	rs607755	Allelic	Fixed	**1.316 (1.029–1.683)**	**0.0284**	0.0661	32.7	0.226	0.397	**1.028–1.683**	0.353	Non-significant
			Dominant	Fixed	**1.520 (1.061–2.178)**	**0.0226**	0.0648	31.5	0.232	0.176	0.810–3.334	0.348	Non-significant
			Heterozygote	Fixed	**1.483 (1.016–2.165)**	**0.0411**	0.0590	7.5	0.339	**0.057**	0.859–2.785	0.811	Non-significant
			Homozygote	Fixed	**1.816 (1.051–3.136)**	**0.0324**	0.0841	40.9	0.184	0.243	**1.030–3.120**	0.320	Non-significant
			Recessive	Fixed	1.317 (0.831–2.086)	0.2411	0.2890	18.6	0.293	0.314	0.818–2.079	0.717	Non-significant
Hernández-García et al. [50]	*RELN*	rs2229864	Allelic	Random	0.809 (0.547–1.198)	0.2896	0.2896	78.3	**0.003**	0.675	0.381–1.715	0.540	Non-significant
			Dominant	Fixed	0.783 (0.500–1.227)	0.2856	0.6264	47.4	0.127	0.186	0.279–2.595	0.114	Non-significant
			Heterozygote	Fixed	0.981 (0.610–1.577)	0.9376	0.9397	0.0	0.547	0.150	0.607–1.588	0.677	Non-significant
			Homozygote	Random	0.772 (0.341–1.744)	0.5334	0.5334	64.6	**0.037**	0.246	0.178–3.324	0.238	Non-significant
			Recessive	Random	0.747 (0.480–1.160)	0.1939	0.1939	73.9	**0.009**	0.903	0.322–1.729	0.547	Non-significant
		rs362691	Allelic	Fixed	0.958 (0.771–1.189)	0.6948	0.6826	6.0	0.378	0.631	0.662–1.355	0.719	Non-significant
			Dominant	Fixed	0.838 (0.580–1.211)	0.3477	0.3352	0.0	0.538	0.255	0.572–1.210	0.746	Non-significant
			Heterozygote	Fixed	0.803 (0.550–1.174)	0.2580	0.2624	0.0	0.559	0.168	0.545–1.180	0.756	Non-significant
			Homozygote	Fixed	1.399 (0.666–2.937)	0.3749	0.4116	0.0	0.720	0.969	0.648–2.821	0.736	Non-significant
			Recessive	Fixed	1.033 (0.773–1.381)	0.8260	0.8336	0.0	0.415	0.744	0.704–1.509	0.715	Non-significant
		rs736707	Allelic	Random	0.975 (0.765–1.243)	0.8391	0.8391	68.8	**0.007**	0.178	0.565–1.682	**0.001**	Non-significant
			Dominant	Random	0.979 (0.696–1.377)	0.9034	0.9034	61.8	**0.023**	0.494	0.472–2.031	**0.001**	Non-significant
			Heterozygote	Random	1.012 (0.819–1.249)	0.9157	0.9576	38.1	0.152	0.513	0.577–1.699	0.713	Non-significant
			Homozygote	Random	0.996 (0.626–1.584)	0.9869	0.9869	62.9	**0.019**	0.178	0.360–2.748	**0.002**	Non-significant
			Recessive	Fixed	1.056 (0.844–1.320)	0.6353	0.8826	36.3	0.165	**0.053**	0.606–1.722	0.723	Non-significant
Wang et al. [40]	*SLC6A4*	5-HTTLPR	Allelic	Random	1.138 (0.849–1.526)	0.3878	0.3878	76.1	**<0.001**	0.511	0.508–2.546	**0.003**	Non-significant
			Dominant	Random	1.201 (0.886–1.644)	0.2337	0.2337	45.1	**0.059**	0.125	0.638–2.248	0.810	Non-significant
			Heterozygote	Random	1.125 (0.776–1.631)	0.5346	0.5346	50.7	**0.032**	**0.035**	0.535–2.244	0.237	Non-significant
			Homozygote	Random	1.358 (0.730–2.525)	0.3341	0.3341	79.7	**<0.001**	0.913	0.268–6.859	0.519	Non-significant
			Recessive	Random	1.110 (0.617–2.000)	0.7274	0.7274	85.7	**<0.001**	0.852	0.186–6.611	**0.025**	Non-significant
Sun [55]	*VDR*	rs11568820	Allelic	Fixed	1.050 (0.893–1.234)	0.5577	0.6390	36.2	0.195	0.964	0.723–1.533	0.054	Non-significant
			Dominant	Fixed	1.028 (0.834–1.266)	0.7969	0.9115	43.1	0.153	0.733	0.605–1.704	**0.037**	Non-significant
			Heterozygote	Fixed	0.992 (0.794–1.240)	0.9445	0.8981	40.1	0.171	0.628	0.584–1.640	0.677	Non-significant
			Homozygote	Fixed	1.118 (0.781–1.600)	0.5435	0.5500	0.0	0.631	0.710	0.779–1.598	0.694	Non-significant
			Recessive	Fixed	1.150 (0.819–1.616)	0.4191	0.4224	0.0	0.843	0.537	0.818–1.615	0.707	Non-significant
		rs1544410	Allelic	Fixed	1.069 (0.923–1.239)	0.3730	0.3734	0.0	0.988	**0.080**	0.923–1.239	0.728	Non-significant
			Dominant	Fixed	1.043 (0.840–1.296)	0.7021	0.7022	0.0	0.824	0.991	0.840–1.296	0.702	Non-significant
			Heterozygote	Fixed	0.996 (0.792–1.252	0.9709	0.9707	0.0	0.451	0.931	0.675–1.475	0.696	Non-significant
			Homozygote	Fixed	1.162 (0.840–1.607)	0.3642	0.3647	0.0	0.894	0.347	0.840–1.604	0.729	Non-significant
			Recessive	Fixed	1.166 (0.894–1.522)	0.2565	0.2619	0.0	0.430	0.535	0.837–1.609	0.159	Non-significant
		rs2228570	Allelic	Fixed	1.002 (0.879–1.143)	0.9736	0.9688	29.5	0.203	0.492	0.804–1.245	0.130	Non-significant
			Dominant	Fixed	0.927 (0.770–1.116)	0.4243	0.4322	30.3	0.197	0.441	0.691–1.226	0.178	Non-significant
			Heterozygote	Fixed	0.873 (0.719–1.060)	0.1712	0.2073	13.9	0.324	0.473	0.710–1.078	0.268	Non-significant
			Homozygote	Random	1.138 (0.714–1.814)	0.5857	0.5857	45.9	**0.085**	0.596	0.494–2.600	0.741	Non-significant
			Recessive	Fixed	1.157 (0.902–1.486)	0.2516	0.3434	30.7	0.193	0.313	0.728–1.854	0.206	Non-significant
		rs731236	Allelic	Fixed	**1.297 (1.125–1.494)**	**0.0003**	**0.0003**	0.0	0.675	0.293	**1.125–1.494**	0.436	**Suggestive**
			Dominant	Fixed	**1.304 (1.082–1.571)**	**0.0053**	**0.0274**	33.3	0.186	0.839	0.897–1.913	0.208	**Weak**
			Heterozygote	Random	1.203 (0.864–1.674)	0.2739	0.2739	60.1	**0.028**	0.933	0.588–2.461	**0.049**	Non-significant
			Homozygote	Fixed	**1.741 (1.258–2.409)**	**0.0008**	**0.0009**	0.0	0.466	0.178	**1.109–2.803**	0.708	**Suggestive**
			Recessive	Fixed	**1.613 (1.187–2.190)**	**0.0022**	**0.0160**	40.2	0.153	0.242	0.807–3.528	0.256	**Weak**
		rs7975232	Allelic	Fixed	**0.823 (0.681–0.993)**	**0.0425**	0.0817	24.4	0.266	0.931	0.587–1.136	0.310	Non-significant
			Dominant	Fixed	0.740 (0.536–1.022)	0.0677	0.0690	0.0	0.614	0.390	0.536–1.024	0.794	Non-significant
			Heterozygote	Fixed	0.759 (0.540–1.066)	0.1118	0.1168	0.0	0.834	**0.014**	0.542–1.071	0.766	Non-significant
			Homozygote	Random	0.528 (0.218–1.276)	0.1558	0.1558	58.3	**0.091**	0.615	0.119–2.306	0.300	Non-significant
			Recessive	Random	0.735 (0.404–1.337)	0.3137	0.3137	65.5	**0.055**	0.663	0.263–2.053	0.152	Non-significant

*PI* Prediction interval.

We found that the rs2710102 polymorphism of *CNTNAP2* was associated with a decreased ASD risk in the allelic (*OR* = 0.849, 95% *CI* = 0.734–0.981, *P* = 0.0263), homozygote (*OR* = 0.668, 95% *CI* = 0.470–0.950, *P* = 0.0248), and recessive (*OR* = 0.715, 95% *CI* = 0.563–0.909, *P* = 0.0062) models. In addition, we found that the mutant allele of rs7794745 (*CNTNAP2*) increased ASD risk based on the dominant (*OR* = 1.300, 95% *CI* = 1.109–1.523, *P* = 0.0012) and heterozygote (*OR* = 1.275, 95% *CI* = 1.081–1.504, *P* = 0.0039) models. The C677T polymorphism of *MTHFR* was associated with an increased ASD risk in the allelic (*OR* = 1.799, 95% *CI* = 1.303–2.483, *P* = 0.0004), dominant (*OR* = 1.959, 95% *CI* = 1.402–2.738, *P* < 0.0001), heterozygote (*OR* = 1.767, 95% *CI* = 1.343–2.330, *P* < 0.0001), and homozygote (*OR* = 1.795, 95% *CI* = 1.158–2.782, *P* = 0.0089) models. The rs607755 polymorphism of *RELN* was associated with an increased ASD risk in the allelic (*OR* = 1.316, 95% *CI* = 1.029–1.683, *P* = 0.0284), dominant (*OR* = 1.520, 95% *CI* = 1.061–2.178, *P* = 0.0226), heterozygote (*OR* = 1.483, 95% *CI* = 1.016–2.165, *P* = 0.0411), and homozygote (*OR* = 1.816, 95% *CI* = 1.051–3.136, *P* = 0.0324) models. The rs731236 polymorphism of *VDR* was associated with an increased ASD risk in the allelic (*OR* = 1.297, 95% *CI* = 1.125–1.494, *P* = 0.0003), dominant (*OR* = 1.304, 95% *CI* = 1.082–1.571, *P* = 0.0053), homozygote (*OR* = 1.741, 95% *CI* = 1.258–2.409, *P* = 0.0008), and recessive (*OR* = 1.613, 95% *CI* = 1.187–2.190, *P* = 0.0022) models. In addition, we found that the mutant allele of rs7975232 (*VDR*) decreased ASD risk (*OR* = 0.823, 95% *CI* = 0.681–0.993, *P* = 0.0425) based on the allelic model. There was no significant association between the other SNPs and ASD risk (all *P* > 0.05; Table 4).

As for the results of *PI*, the null value was excluded in only four SNPs of rs2710102 (*CNTNAP2*) under the allelic, homozygote, and recessive models; rs7794745 (*CNTNAP2*) under the heterozygote model; rs607755 (*RELN*) and rs731236 (*VDR*) under the allelic and homozygote models (Table 4). When evaluating small-study effects using Egger’s regression asymmetry test, evidence for statistically significant small-study effects in the meta-analyses was identified in some SNPs. Supporting evidence included a meta-analysis on A1298C (*MTHFR*) under the allelic, dominant, and heterozygote models; a meta-analysis on C677T (*MTHFR*) under the five genetic models; a meta-analysis on rs20317 (*GABRB3*) under the dominant and heterozygote models; one each on rs736707 (*RELN*) and rs1544410 (*VDR*) under the recessive and allelic models, respectively; and three meta-analyses on rs607755 (*RELN*), 5-HTTLPR (*SLC6A4*), and rs7975232 (*VDR*) under the heterozygote model (*P* < 0.10).

Hints of excess-statistical-significance bias were observed in rs2710102 (*CNTNAP2*) under the allelic, homozygote, and recessive models; rs4680 (*COMT*) under the allelic model; rs20317 (*GABRB3*) under the heterozygote model; A1298C (*MTHFR*) under allelic, dominant, heterozygote, and recessive models; C677T (*MTHFR*) under homozygote and recessive models; rs736707 (*RELN*) under allelic, dominant, and homozygote models; 5-HTTLPR (*SLC6A4*) under allelic and recessive models; rs11568820 (*VDR*) under the dominant model; and rs731236 (*VDR*) under the heterozygote model, with statistically significant (*P* < 0.05) excess of positive studies (Table 4).

We categorized the strength of the evidence of 20 SNPs for ASD into five levels. According to the criteria for the level of evidence, for rs2710102 (*CNTNAP2*), the *P*-value based on the random effects model was significant at *P* < 0.05 under allelic, homozygote, and recessive models. Between-study heterogeneity was not significant (*P* > 0.10, *I²* < 50.0%), the 95% *PI* did not exclude the null value, and there was no excess significance bias (*P* > 0.05) under the five genetic models. For rs7794745 (*CNTNAP2*), the *P*-value based on the random effects model was significant at *P* < 0.05 under dominant and heterozygote models. For C677T (*MTHFR*), there was a total of 2147 ASD cases, which was > 1000, and the *P*-value based on the random effects model was significant at *P* < 10^–3^ under allelic, dominant, and heterozygote models. Moreover, it was significant at *P* < 0.05 under the homozygote model. Between-study heterogeneity was large (*I²* > 50.0%) under the five genetic models, the 95% *PI* did not exclude the null value under the five genetic models, and there was no excess significance bias (*P* > 0.05) under allelic, dominant, and heterozygote models. For rs731236 (*VDR*), there was a total of 1088 ASD cases, which was >1000, the *P*-value based on the random effects model was significant at *P* < 10^–3^ under allelic and homozygote models, and the *P*-value was significant at *P* < 0.05 under dominant and recessive models. Between-study heterogeneity was not significant (*P* > 0.10, *I²* < 50.0%), the 95% *PI* excluded the null value, and there was no small-study effect (*P* > 0.10) and excess significance bias (*P* > 0.05) under the five genetic models (Table 4). Thus, the rs2710102 (*CNTNAP2*) was graded as weak evidence (class IV) under allelic, homozygote, and recessive models; rs7794745 (*CNTNAP2*) was graded as weak evidence (class IV) under dominant and heterozygote models; the C677T (*MTHFR*) was graded as suggestive evidence (class III) under allelic, dominant, and heterozygote models; C677T (*MTHFR*) was graded as weak evidence (class IV) under the homozygote model; *VDR* (rs731236) was graded as suggestive evidence (class III) under allelic and homozygote models; and *VDR* (rs731236) was graded as weak evidence (class IV) under dominant and recessive models.

## Discussion

This UR summarizes evidence on the genetic basis of ASD. Our study design provides a robust and significant synthesis of published evidence and increases the conclusive power with more precise estimates. Overall, 12 significant SNPs of *CNTNAP2*, *MTHFR*, *OXTR*, *SLC25A12*, and *VDR* were identified from 41 SNPs of nine candidate genes in 28 meta-analyses. Of those, associations with suggestive evidence (class III) were the C677T polymorphism of *MTHFR* (under allelic, dominant, and heterozygote models) and rs731236 polymorphism of *VDR* (under allelic and homozygote models). Associations with weak evidence (class IV) were the rs2710102 polymorphism of *CNTNAP2* (under allelic, homozygote, and recessive models), rs7794745 polymorphism of *CNTNAP2* (under dominant and heterozygote models), C677T polymorphism of *MTHFR* (under homozygote model), and rs731236 polymorphism of *VDR* (under dominant and recessive models).

ASD remains a ‘disease of theories’, as multiple genes and environmental risk factors are probably involved in its pathogenesis. However, to date, the etiology and pathological mechanism of ASD are still unknown [57]. The genetic architecture of ASD is complex. Moreover, most research in this field has focused on candidate genes, primarily those with a plausible role in the known underlying pathophysiology, including mitochondrial dysfunction, abnormal neurodevelopment, and dysfunction of synapse formation and stability during neurodevelopment [58, 59].

*CNTNAP2* is a member of neurexin superfamily and is a synaptic protein [60]. It plays a major role in neural development, crucial for neural circuit assembly [61]. *CNTNAP2* mutations may be linked to the abnormal behavior of ASD by altering synaptic neurotransmission, functional connectivity, and neuronal network activity [61, 62]. The rs2710102 and rs7794745 are two common non-coding variants in *CNTNAP2*, with four and three meta-analyses reporting the associations with ASD, respectively. The results of the meta-analysis by Uddin et al. were inconsistent with the other authors’ [44]. We further re-analyzed and categorized the strengths of evidence. Both the rs2710102 and rs7794745 polymorphisms of *CNTNAP2* were associated with decreased risk of ASD. The rs2710102 was graded as having a weak association with ASD under allelic, homozygote, and recessive models. The rs7794745 was graded as having a weak association with ASD under dominant and heterozygote models. Therefore, it is likely that the rs2710102 and rs7794745 polymorphisms of *CNTNAP2* influence the risk of ASD.

*MTHFR* is one of the most frequently-researched genes in ASD, with four and eight meta-analyses for A1298C [29, 31–33] and C667T [29–36] polymorphisms, respectively. The A1298C and C667T polymorphisms of *MTHFR* are associated with reduced enzymatic activity, which affects folate metabolism, and, consequently, fetal brain development [29, 32, 33]. Dysfunction of the brain is indicated in ASD etiology; thus, *MTHFR* has been the focal point of investigation in this disorder. The meta-analysis by Li et al. was selected because it was the most recent among the examined meta-analyses [34]. The genotype distributions of the A1298C and C667T polymorphisms of *MTHFR* in the control group were not found in the HWE, which may be due to selection bias, population stratification, and genotyping errors within the original studies. We found no significant association between the A1298C polymorphism of *MTHFR* and ASD risk in the five genetic models, which was consistent with the four meta-analyses, indicating that the A1298C polymorphism of *MTHFR* may not be a risk SNP of ASD. We found that the C667T polymorphism of *MTHFR* was associated with an increased risk of ASD, graded as having suggestive association under allelic, dominant, and heterozygote models and weak association under the homozygote model. Thus, the C667T polymorphism of *MTHFR* may confer ASD risk.

*OXTR, a* neuropeptide gene, is also one of the most frequently-studied genes associated with ASD [45]. Oxytocin plays an important role in a range of human behaviors, including affiliative behavior to social bonding, and is differentially expressed in the blood of individuals with autism compared to that of non-autistic individuals [45, 63]. Three meta-analyses investigated 19 SNPs and ASD risk. Of these, only rs2254298 and rs53576 were analyzed in two meta-analyses [45, 46], and the remaining SNPs were unique in one meta-analysis. Three SNPs (rs2268491, rs237887, and rs7632287) were significantly associated with ASD risk [45, 46]; however, we failed to determine the credibility of the evidence because of the lack of original data.

*RELN* encodes a large secreted extracellular matrix protein considered to be involved in neuronal migration, brain structure construction, synapse formation, and stability during neurodevelopment [59]. Fatemi et al. found decreased levels of reelin mRNA and protein and increased levels of reelin receptors in the brain and plasma of individuals with autism [64]. Dysfunction of the reelin signaling pathway has been found in ASD, schizophrenia, epilepsy, bipolar disorder, mental retardation, depression, Alzheimer’s disease, and lissencephaly [59, 65]. Genetic association studies have been conducted to investigate the associations between SNPs within *RELN* and ASD with conflicting results. None of the three meta-analyses found significant associations [48–50]. The meta-analysis by Hernández-García et al. was retained for further analysis of the original studies after comparing publication years and sample sizes of the three meta-analyses [50]. Hernández-García et al. did not find a significant association between *RELN* and ASD risk [50]. In our analysis, because there was no substantial statistical heterogeneity under the five genetic models (all *P* > 0.10, *I*^*2*^ ≤ 50%), a fixed model was applied to pool the effect size. We found that the rs607755 of *RELN* was associated with ASD risk in allelic, dominant, heterozygote, and homozygote models. This inconsistent result was caused by different pooling methods, indicating that it is necessary to perform an UR to provide a robust synthesis of published evidence and evaluate the importance of genetic factors related to ASD. Our UR results showed that the rs607755 of *RELN* was not significant when we categorized the strength of the evidence. Thus, it may not be a risk factor for ASD.

*SLC25A12* encodes the mitochondrial aspartate/glutamate carrier of the brain, a calcium-binding solute carrier located in the inner mitochondrial membrane that is expressed principally in the heart, brain, and skeletal muscle [66, 67]. Rossignol et al. found that individuals with ASD had a significantly higher prevalence of mitochondrial diseases than that of controls, indicating the involvement of mitochondrial dysfunction in ASD [58]. Thus, an increasing number of genetic studies on ASD have focused on *SLC25A12*. However, the results on the association between SNPs of *SLC25A12* and ASD risk are inconsistent. Two meta-analyses were performed by Aoki et al. [53] and Liu et al. [54], and despite differences in the number of studies between the two meta-analyses, both found a higher risk of ASD in individuals with the mutant allele of rs2056202 or rs2292813. However, we failed to determine the credibility of the evidence because of a lack of original data.

Vitamin D plays a significant role in brain homeostasis, neurodevelopment, and immunological modulation, and its deficiency has been reported in children with ASD [68]. Hence, changes in the genes involved in the transport or binding of vitamin D may be associated with ASD risk. Notably, vitamin D exerts its effects on genes via the *VDR* gene, to which changes may be an underlying risk factor for ASD. Sun et al. [55] and Yang et al. [56] performed meta-analyses to pool the effect size of inconsistent conclusions from original studies on the associations between SNPs in *VDR* and ASD risks. We further re-analyzed and categorized the strengths of evidence. The rs731236 polymorphism of *VDR* was associated with an increased risk of ASD, graded as having a suggestive association under allelic and homozygote models and a weak association under dominant and recessive models without small-study effects, excess significance bias, and large heterogeneity. It is likely that the *VDR* rs731236 polymorphism influences the risk of ASD.

Our study has some limitations. First, associations between several SNPs and ASD risks under five genetic models or in different populations were not fully assessed in our UR, partly due to insufficient original data. Second, our UR is limited by significant heterogeneity that may be caused by population stratification, study design, and differences in the pattern of linkage disequilibrium structure. Finally, ASD is a complex disorder with different causative factors (multiple genetic and environmental factors). We did not investigate the involvement of environmental factors in ASD. Despite these limitations above, our UR includes its prospective registration with PROSPERO, an extensive search strategy, clear criteria of inclusion and exclusion, duplicated processing by two authors, accurate quality assessment, systematic assessment and critical comparison of meta-analyses, and consistent standards for re-analysis of original data.

In conclusion, our UR summarizes evidence on the genetics of ASD and provides a broad and detailed overview of risk genes for ASD. The rs2710102 and rs7794745 polymorphisms of *CNTNAP2*, C677T polymorphism of *MTHFR*, and rs731236 polymorphism of *VDR* may confer ASD risk. This study will aid clinicians in decision-making through the use of evidence-based information on the most salient candidate genes relevant to ASD and recommendations for future treatment, prevention, and research.

## References

[1] Lai MC, Lombardo MV, Baron-Cohen S (2014). Autism. Lancet.

[2] WHO Questions and answers about autism spectrum disorders (ASD). 2021; http://www.who.int/features/qa/85/en/. Accessed 5 July 2021.

[3] Baxter AJ, Brugha TS, Erskine HE, Scheurer RW, Vos T, Scott JG (2015). The epidemiology and global burden of autism spectrum disorders. Psychological Med.

[4] Chaste P, Leboyer M (2012). Autism risk factors: genes, environment, and gene-environment interactions. Dialogues Clin Neurosci.

[5] Kim JY, Son MJ, Son CY, Radua J, Eisenhut M, Gressier F (2019). Environmental risk factors and biomarkers for autism spectrum disorder: an umbrella review of the evidence. lancet Psychiatry.

[6] Lord C, Brugha TS, Charman T, Cusack J, Dumas G, Frazier T (2020). Autism spectrum disorder. Nat Rev Dis Prim.

[7] Ronald A, Hoekstra RA (2011). Autism spectrum disorders and autistic traits: a decade of new twin studies. Am J Med Genet Part B, Neuropsychiatr Genet: Off Publ Int Soc Psychiatr Genet.

[8] Sandin S, Lichtenstein P, Kuja-Halkola R, Larsson H, Hultman CM, Reichenberg A (2014). The familial risk of autism. JAMA.

[9] Peñagarikano O, Abrahams BS, Herman EI, Winden KD, Gdalyahu A, Dong H (2011). Absence of CNTNAP2 leads to epilepsy, neuronal migration abnormalities, and core autism-related deficits. Cell.

[10] Qiu S, Li Y, Bai Y, Shi J, Cui H, Gu Y (2019). SHANK1 polymorphisms and SNP-SNP interactions among SHANK family: a possible cue for recognition to autism spectrum disorder in infant age. Autism Res.

[11] Grove J, Ripke S, Als TD, Mattheisen M, Walters RK, Won H (2019). Identification of common genetic risk variants for autism spectrum disorder. Nat Genet.

[12] Ioannidis JP (2009). Integration of evidence from multiple meta-analyses: a primer on umbrella reviews, treatment networks and multiple treatments meta-analyses. CMAJ: Can Med Assoc J = J de l’Assoc Med Canadienne.

[13] van der Burg NC, Al Hadithy AFY, van Harten PN, van Os J, Bakker PR (2020). The genetics of drug-related movement disorders, an umbrella review of meta-analyses. Mol Psychiatry.

[14] Shea BJ, Hamel C, Wells GA, Bouter LM, Kristjansson E, Grimshaw J (2009). AMSTAR is a reliable and valid measurement tool to assess the methodological quality of systematic reviews. J Clin Epidemiol.

[15] Giannakou K, Evangelou E, Papatheodorou SI (2018). Genetic and non-genetic risk factors for pre-eclampsia: umbrella review of systematic reviews and meta-analyses of observational studies. Ultrasound Obstet Gynecol.

[16] Yang T, Li X, Montazeri Z, Little J, Farrington SM, Ioannidis JPA (2019). Gene-environment interactions and colorectal cancer risk: an umbrella review of systematic reviews and meta-analyses of observational studies. Int J Cancer.

[17] Lau J, Ioannidis JP, Schmid CH (1997). Quantitative synthesis in systematic reviews. Ann Intern Med.

[18] Higgins JP, Thompson SG, Spiegelhalter DJ (2009). A re-evaluation of random-effects meta-analysis. J Roy Soc Statistical Soc A (Stat Soc).

[19] Higgins JP (2008). Commentary: Heterogeneity in meta-analysis should be expected and appropriately quantified. Int J Epidemiol.

[20] Cochran WGJB (1954). Combination Estimates Differ Exp.

[21] Higgins JP, Thompson SG (2002). Quantifying heterogeneity in a meta-analysis. Stat Med.

[22] Ioannidis JP, Patsopoulos NA, Evangelou E (2007). Uncertainty in heterogeneity estimates in meta-analyses. BMJ (Clin Res Ed).

[23] Sterne JA, Sutton AJ, Ioannidis JP, Terrin N, Jones DR, Lau J (2011). Recommendations for examining and interpreting funnel plot asymmetry in meta-analyses of randomised controlled trials. BMJ (Clin Res Ed).

[24] Egger M, Davey Smith G, Schneider M, Minder C (1997). Bias in meta-analysis detected by a simple, graphical test. Clin Focus.

[25] Ioannidis JPA (2013). Clarifications on the application and interpretation of the test for excess significance and its extensions. J Math Psychol.

[26] Belbasis L, Köhler CA, Stefanis N, Stubbs B, van Os J, Vieta E (2018). Risk factors and peripheral biomarkers for schizophrenia spectrum disorders: an umbrella review of meta-analyses. Acta Psychiatr Scand.

[27] Bellou V, Belbasis L, Tzoulaki I, Evangelou E, Ioannidis JP (2016). Environmental risk factors and Parkinson’s disease: an umbrella review of meta-analyses. Parkinsonism Relat Disord.

[28] Belbasis L, Bellou V, Evangelou E, Ioannidis JP, Tzoulaki I (2015). Environmental risk factors and multiple sclerosis: an umbrella review of systematic reviews and meta-analyses. Lancet Neurol.

[29] Pu D, Shen Y, Wu J (2013). Association between MTHFR gene polymorphisms and the risk of autism spectrum disorders: a meta-analysis. Autism Res.

[30] Rai V (2016). Association of methylenetetrahydrofolate reductase (MTHFR) gene C677T polymorphism with autism: evidence of genetic susceptibility. Metab Brain Dis.

[31] Sadeghiyeh T, Dastgheib SA, Mirzaee-Khoramabadi K, Morovati-Sharifabad M, Akbarian-Bafghi MJ, Poursharif Z (2019). Association of MTHFR 677C>T and 1298A>C polymorphisms with susceptibility to autism: a systematic review and meta-analysis. Asian J Psychiatry.

[32] Razi B, Imani D, Hassanzadeh Makoui M, Rezaei R, Aslani S (2020). Association between MTHFR gene polymorphism and susceptibility to autism spectrum disorders: systematic review and meta-analysis. Res Autism Spectrum Disorders.

[33] Li Y, Qiu S, Shi J, Guo Y, Li Z, Cheng Y (2020). Association between MTHFR C677T/A1298C and susceptibility to autism spectrum disorders: a meta-analysis. BMC Pediatrics.

[34] Li CX, Liu YG, Che YP, Ou JL, Ruan WC, Yu YL (2021). Association between MTHFR C677T polymorphism and susceptibility to autism spectrum disorders: a meta-analysis in Chinese Han population. Front Pediatrics.

[35] Wang S, Wu J (2021). Association between MTHFR gene C677T polymorphism and risk of autism spectrum disorder in children: a Meta-analysis. Chin J Obstet Gynecol Pediatr.

[36] Zhang Y, Gai C, Yang L, Ma H, Zhang J, Sun H (2021). Meta-analysis of the relationship between MTHFR C677T gene polymorphism and susceptibility to autism spectrum disorders. Tianjin Med J.

[37] Huang CH, Santangelo SL (2008). Autism and serotonin transporter gene polymorphisms: a systematic review and meta-analysis. Am J Med Genet B: Neuropsychiatr Genet.

[38] Mo S, Qi X, Shao S, Sun Z, Song R (2013). An integrated meta-analysis of the association between 5-HTTLPR and autism spectrum disorder. Acta Med Univ Sci Technol Huazhong.

[39] Yang PY, Menga YJ, Li T, Huang Y (2017). Associations of endocrine stress-related gene polymorphisms with risk of autism spectrum disorders: evidence from an integrated meta-analysis. Autism Res.

[40] Wang H, Yin F, Gao J, Fan X (2019). Association between 5-HTTLPR polymorphism and the risk of autism: a meta-analysis based on case-control studies. Front psychiatry.

[41] Werling AM, Bobrowski E, Taurines R, Gundelfinger R, Romanos M, Grünblatt E (2016). CNTNAP2 gene in high functioning autism: no association according to family and meta-analysis approaches. J Neural Transm.

[42] Zhang T, Zhang J, Wang Z, Jia M, Lu T, Wang H (2019). Association between CNTNAP2 polymorphisms and autism: a family-based study in the chinese han population and a meta-analysis combined with GWAS data of psychiatric genomics consortium. Autism Res.

[43] Wang Y, Liu Y, Xia Z, Yu H, Gai Z (2019). Association of the contactin-association protein-like 2 gene rs2710102 polymorphism and autism spectrum disorders: a meta-analysis. Clin Focus.

[44] Uddin MS, Azima A, Aziz MA, Aka TD, Jafrin S, Millat MS (2021). CNTNAP2 gene polymorphisms in autism spectrum disorder and language impairment among Bangladeshi children: a case-control study combined with a meta-analysis. Hum Cell.

[45] LoParo D, Waldman ID (2015). The oxytocin receptor gene (OXTR) is associated with autism spectrum disorder: a meta-analysis. Mol Psychiatry.

[46] Kranz TM, Kopp M, Waltes R, Sachse M, Duketis E, Jarczok TA (2016). Meta-analysis and association of two common polymorphisms of the human oxytocin receptor gene in autism spectrum disorder. Autism Res.

[47] Zhou J. Association between the single nucleotide polymorphism (SNP) of oxytocin receptor (OXTR) gene and Autism Spectrum Disorders (ASD): a meta-analysis. Jining Medical University. 2020.

[48] Wang Z, Hong Y, Zou L, Zhong R, Zhu B, Shen N (2014). Reelin gene variants and risk of autism spectrum disorders: an integrated meta-analysis. Am J Med Genet Part B: Neuropsychiatr Genet.

[49] Chen N, Bao Y, Xue Y, Sun Y, Hu D, Meng S (2017). Meta-analyses of RELN variants in neuropsychiatric disorders. Behav Brain Res.

[50] Hernández-García I, Chamorro AJ, de la Vega HGT, Carbonell C, Marcos M, Mirón-Canelo JA (2020). Association of allelic variants of the reelin gene with autistic spectrum disorder: A systematic review and meta-analysis of candidate gene association studies. Int J Environ Res Public Health.

[51] Mahdavi M, Kheirollahi M, Riahi R, Khorvash F, Khorrami M, Mirsafaie M (2018). Meta-analysis of the association between GABA receptor polymorphisms and autism spectrum disorder (ASD). J Mol Neurosci.

[52] Noroozi R, Taheri M, Ghafouri-Fard S, Bidel Z, Omrani MD, Moghaddam AS (2018). Meta-analysis of GABRB3 gene polymorphisms and susceptibility to autism spectrum disorder. J Mol Neurosci.

[53] Aoki Y, Cortese S (2016). Mitochondrial aspartate/glutamate carrier SLC25A12 and autism spectrum disorder: a meta-analysis. Mol Neurobiol.

[54] Liu J, Yang A, Zhang Q, Yang G, Yang W, Lei H (2015). Association between genetic variants in SLC25A12 and risk of autism spectrum disorders: An integrated meta-analysis. Am J Med Genet Part B: Neuropsychiatr Genet.

[55] Sun J. Association between vitamin D receptor gene polymorphism and susceptibility to autism spectrum disorders: a meta-analysis. Jining Medical University. 2020.

[56] Yang H, Wu X (2020). The correlation between vitamin D receptor (VDR) gene polymorphisms and autism: a meta-analysis. J Mol Neurosci.

[57] Kojic M, Gawda T, Gaik M, Begg A, Salerno-Kochan A, Kurniawan ND (2021). Elp2 mutations perturb the epitranscriptome and lead to a complex neurodevelopmental phenotype. Nat Commun.

[58] Rossignol DA, Frye RE (2012). Mitochondrial dysfunction in autism spectrum disorders: a systematic review and meta-analysis. Mol psychiatry.

[59] Jossin Y. Reelin functions, mechanisms of action and signaling pathways during brain development and maturation. Biomolecules. 2020;10:964. 10.3390/biom10060964 PMC7355739 32604886

[60] Arking DE, Cutler DJ, Brune CW, Teslovich TM, West K, Ikeda M (2008). A common genetic variant in the neurexin superfamily member CNTNAP2 increases familial risk of autism. Am J Hum Genet.

[61] Lazaro MT, Taxidis J, Shuman T, Bachmutsky I, Ikrar T, Santos R (2019). Reduced prefrontal synaptic connectivity and disturbed oscillatory population dynamics in the CNTNAP2 model of autism. Cell Rep.

[62] Toma C, Pierce KD, Shaw AD, Heath A, Mitchell PB, Schofield PR (2018). Comprehensive cross-disorder analyses of CNTNAP2 suggest it is unlikely to be a primary risk gene for psychiatric disorders. PLoS Genet.

[63] Modahl C, Green L, Fein D, Morris M, Waterhouse L, Feinstein C (1998). Plasma oxytocin levels in autistic children. Biol psychiatry.

[64] Fatemi SH (2005). Reelin glycoprotein: structure, biology and roles in health and disease. Mol psychiatry.

[65] Fatemi SH (2005). Reelin glycoprotein in autism and schizophrenia. Int Rev Neurobiol.

[66] Silverman JM, Buxbaum JD, Ramoz N, Schmeidler J, Reichenberg A, Hollander E (2008). Autism-related routines and rituals associated with a mitochondrial aspartate/glutamate carrier SLC25A12 polymorphism. Am J Med Genet B: Neuropsychiatr Genet.

[67] Anitha A, Nakamura K, Thanseem I, Yamada K, Iwayama Y, Toyota T (2012). Brain region-specific altered expression and association of mitochondria-related genes in autism. Mol Autism.

[68] Saad K, Abdel-Rahman AA, Elserogy YM, Al-Atram AA, Cannell JJ, Bjørklund G (2016). Vitamin D status in autism spectrum disorders and the efficacy of vitamin D supplementation in autistic children. Nutritional Neurosci.

